# Haplotype-assisted accurate non-invasive fetal whole genome recovery through maternal plasma sequencing

**DOI:** 10.1186/gm422

**Published:** 2013-02-27

**Authors:** Shengpei Chen, Huijuan Ge, Xuebin Wang, Xiaoyu Pan, Xiaotian Yao, Xuchao Li, Chunlei Zhang, Fang Chen, Fuman Jiang, Peipei Li, Hui Jiang, Hancheng Zheng, Lei Zhang, Lijian Zhao, Wei Wang, Songgang Li, Jun Wang, Jian Wang, Huanming Yang, Yingrui Li, Xiuqing Zhang

**Affiliations:** 1 BGI-Shenzhen, Shenzhen 518083, China; 2 State Key Laboratory of Bioelectronics, School of Biological Science and Medical Engineering, Southeast University, Nanjing 210096, China; 3 School of Bioscience and Bioengineering, South China University of Technology, Guangzhou 510000, China

## Abstract

**Background:**

The applications of massively parallel sequencing technology to fetal cell-free DNA (cff-DNA) have brought new insight to non-invasive prenatal diagnosis. However, most previous research based on maternal plasma sequencing has been restricted to fetal aneuploidies. To detect specific parentally inherited mutations, invasive approaches to obtain fetal DNA are the current standard in the clinic because of the experimental complexity and resource consumption of previously reported non-invasive approaches.

**Methods:**

Here, we present a simple and effective non-invasive method for accurate fetal genome recovery-assisted with parental haplotypes. The parental haplotype were firstly inferred using a combination strategy of trio and unrelated individuals. Assisted with the parental haplotype, we then employed a hidden Markov model to non-invasively recover the fetal genome through maternal plasma sequencing.

**Results:**

Using a sequence depth of approximately 44X against a an approximate 5.69% cff-DNA concentration, we non-invasively inferred fetal genotype and haplotype under different situations of parental heterozygosity. Our data show that 98.57%, 95.37%, and 98.45% of paternal autosome alleles, maternal autosome alleles, and maternal chromosome X in the fetal haplotypes, respectively, were recovered accurately. Additionally, we obtained efficient coverage or strong linkage of 96.65% of reported Mendelian-disorder genes and 98.90% of complex disease-associated markers.

**Conclusions:**

Our method provides a useful strategy for non-invasive whole fetal genome recovery.

## Background

Prenatal diagnosis is one of the most efficient approaches to decrease the incidence of birth defects [1]. Traditionally, fetal cells for prenatal diagnosis are collected invasively by the procedures of amniocentesis or chorionic villus sampling (CVS), but these carry a risk of miscarriage [2,3]. To reduce the requirement of invasive testing, non-invasive approaches, such as the use of maternal serum markers and ultrasound, are widely used in the clinic to classify low- and high-risk pregnant women with Down's syndrome fetuses. However, these non-invasive prenatal screens are unsatisfactory to many clinicians and pregnant women due to their false-positive and potential false-negative rates [4-6]. With the discovery of cell-free fetal DNA (cff-DNA) in maternal plasma [7-10] and the emergence of high-throughput sequencing, the clinical application of non-invasive tests to detect fetal chromosomal abnormalities using maternal plasma sequencing have been discussed [11-13].

Theoretically, it should be possible to recover the fetal genome non-invasively through maternal plasma sequencing to enable the comprehensive prenatal diagnosis of Mendelian diseases and lessen the need for invasive procedures [14,15]. In 2010, Lo's group showed the feasibility of non-invasive fetal whole genome recovery and inferring the fetal genotype, although they did not assess the biparentally heterozygous sites in the fetal genome [14]. Recent studies from Kitzman *et al. *[15] and Fan *et al. *[16] introduced accurate non-invasive fetal genotype inference methods assisted by maternal haplotype, but their methods showed uncertain performance in detecting paternal transition in low cff-DNA concentrations. In early gestation, the concentration of cff-DNA is approximately 3% to 6% of the total cell-free DNA [17], which may lead to uneven recovery of the paternal allele in the whole genome. Robust strategies of noninvasively detecting both maternal and paternal alleles are still needed. Moreover, the fetal haplotype information is especially useful in detecting some haplotype-related diseases, such as systemic lupus erythematosus [18], as well as personal genomic analyses in the future [19,20].

Here, we developed a novel strategy of fetal genome recovery, inferring the fetal genotype as well as haplotype at the same time. Given the fact that the fetal genome is the combination of parentally transmitted chromosomes, we reconstructed the fetal genome by observing parental allele transition in maternal plasma. We first used a combined strategy of trios and unrelated individuals to construct parental haplotypes, and then observed the parental allele transition in maternal plasma and optimized the fetal haplotype using a hidden Markov model (HMM) and Viterbi algorithm. Thereby, we recovered the fetal haplotype as well as the genotype against all parental heterozygosity in one step. Our method highlights the prospective value to translational medicine of non-invasive prenatal diagnosis to recover the fetal genome using maternal plasma sequencing.

## Methods

### Sample preparation

In this study, a Chinese couple and both parents of the couple were recruited with written informed consent. Also, this study was approved by the institutional review board of BGI-Shenzhen and conducted in accordance with the Declaration of Helsinki.

#### Peripheral blood

We collected 10 mL of peripheral blood from a woman with pregnancy of 13 weeks of gestation, 5 mL of peripheral blood from her husband, and 10 mL of fetal umbilical blood after the delivery. Blood samples from each participant were collected in EDTA-containing tubes.

#### Maternal plasma

We obtained maternal plasma from 10 mL maternal peripheral blood after centrifugation at 1,600 *g *for 10 min. Great care was taken in the collection of plasma samples to avoid taking the buffy coat or any blood clots. Plasma was transferred to 2.0 mL eppendorf tubes and centrifuged at 16,000 *g *for 10 min to remove residual cells. Blood samples and plasma samples were stored at -20°C and -80°C, respectively, until further processing.

#### Saliva

Saliva was collected from grandparents using Oragene ^® ^OG-250 tubes and kits, following the standard manufacturer's instructions.

### DNA extraction

g-DNA from whole blood, saliva, and maternal plasma were extracted by using a TIANamp Micro DNA Kit (Tiangen) according to the manufacturer's instructions.

### Library preparation and massively parallel genomic sequencing

#### Genomic DNA

One microgram of g-DNA was sheared by an S2 sonicator (Covaris, Inc.), yielding fragments between 100 and 500 bp, with a predominance of 300 bp. For massively parallel genomic sequencing, approximately 1 μg of fragmented g-DNA was prepared for library construction. Briefly, DNA fragments were blunt-ended using T4 DNA polymerase (Enzymatics), Klenow polymerase (Enzymatics), and T4 polynucleotide kinase (Enzymatics) and were ligated to adapters after addition of terminal A nucleotides. The adapter-ligated DNA fragments in the range of 300 to 350 bp were size-selected using 2% agarose electrophoresis and then amplified using a 10-cycle PCR. An Agencourt AMPure 450 mL Kit was used for the purification of PCR products.

#### Plasma DNA

Plasma DNA (10 to 50 ng) was used for library preparation according to a modified protocol, in which a 17-cycle PCR was conducted to enrich adapter-ligated DNA fragments.

#### Library QC and sequencing

The libraries were quality-controlled by using an Agilent DNA 1000 kit on the 2100 Bioanalyzer (Agilent) platform and quantified by real-time PCR. DNA libraries were hybridized to the surface of sequencing flowcells, and DNA clusters were generated after amplification. The libraries were then sequenced using the Illumina Hiseq™ 2000 sequencing system according to the manufacturer's instructions. The sequence reads of this parent-offspring trio have been uploaded to the NCBI SRA database (SRA060043).

### Illumina DNA microarray

The construction of the library and scanning of the microarray (Omni 2.5 SNP-array) were done according to the manufacturer's instructions for the corresponding array and for Iscan.

### Bioinformatics

Bioinformatic analyses are described in the Additional file 1 (Additional file 1, Supplementary Methods).

## Results

### Accurate fetal genome recovery through maternal plasma

**Figure 1:**
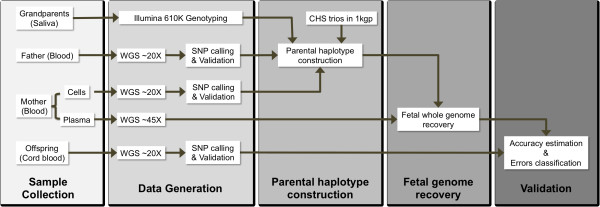
**The research principle of our study**. To recover the fetal genome, we divided our work into several parts. We first recruited a family that included three entire generations. The parental genotypes were determined by whole genome sequencing, whereas the grandparents' were determined by SNP array. We then constructed parental haplotypes with a combined trio and unrelated-individual strategy. Assisted by the parental haplotypes, we successfully recovered the fetal genome via maternal plasma DNA sequencing. Finally, we performed a validation using the child's cord blood after the delivery.

To perform haplotype-assisted accurate non-invasive fetal whole genome recovery through maternal plasma sequencing (Figure 1), we recruited a Chinese woman with pregnancy of 13 weeks of gestation and her family, including three generations, as well as fetal blood after delivery. We then performed approximately 44X and 20X whole genome shotgun sequencing of the plasma sample and of parental genomic DNA (g-DNA), respectively (Table 1). The cff-DNA concentration of this male fetus was estimated as 5.69% using the biparentally homozygous sites. Illumina Infinium HD Human610-Quad BeadChip was used to genotype the gDNA from grandparents to construct the parental haplotypes. Also, we used Illumina HumanOmni2.5-8 BeadChip to validate the accuracy of parental SNP calling, in which the parental genotypes were validated as approximately 99.22% consistent with the array (Table 1 and Additional file 1, Table S1).

**Table 1:** Data production

Microarray array							
**Samples**	**Type of DNA**	**Type of microarray**		**Call rate (%)**		**SNP calling (*n*) (10^5^)**	

Grandparents^a^	g-DNA (saliva)	Human 610-Quad BeadChip		99.70 ± 0.07		5.89 ± 0.004	

**WGS**							

**Samples**	**Type of DNA**	**Reads (*n*) (10^9^)**	**Production (Gb)**	**Map rate (%)**	**Coverage (%)**	**Depth (fold)**	**Consistency in validation (%)**

Father	g-DNA (blood)	0.72	71.89	89.75	99.71	21.86	99.23
Mother	g-DNA (blood)	0.74	74.03	90.19	99.09	20.96	99.19
Offspring	g-DNA (cord blood)	0.72	72.17	90.64	99.75	21.32	99.25
Plasma	Plasma DNA	1.81	179.63	83.68	99.47	43.91	-

^a^Mean ± standard deviation.

We then performed a parental haplotype construction with a combined strategy of trios and unrelated individuals. Although both trios and unrelated individuals could be applied to construct the parental haplotypes, the haplotype ambiguity in trio strategy and the stratification in unrelated individual strategy would significantly restrict the value of either of these strategies. In this study, the parental haplotypes were obtained by BEAGLE [21] using their sequencing genotype and the genotyping data of the grandparents along with the newly released 51 parent-offspring trios of Chinese Han in the 1000 Genomes project (pilot II). By using this strategy, the inferred rate of parental haplotypes increased, on average, from 90.32% to 100% compared to using a trio strategy only.

**Figure 2:**
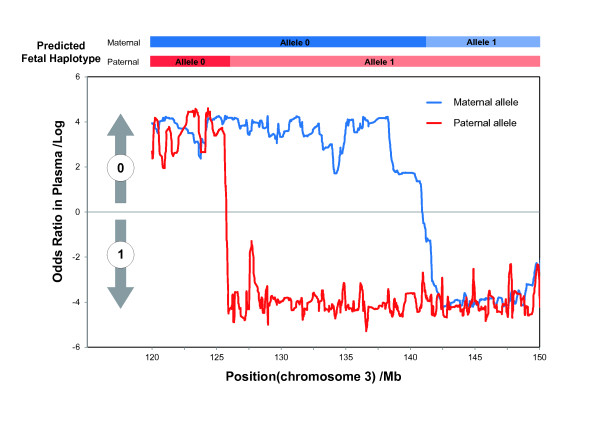
**Identification of recombination breakpoints by HMM**. This figure shows the HMM-based detection of recombination and the predicted fetal haplotype. A genomic region from on Chr3 (120-150 Mb) is shown with lines (red for paternal allele, blue for maternal allele) indicating the logarithmic odds ratio between transmission probability of haplotype 1 and haplotype 0, which were computed by the HMM at each site. The color-coded chart (top) shows the predicted fetal haplotype as a combination of parental alleles.

Assisted by the parental haplotypes, we then developed an efficient method for fetal whole genome recovery through maternal plasma sequencing. Ideally, in maternal plasma sequencing with a site-by-site strategy (SBSS), we could reconstruct the paternally transmitted allele directly by determining the nucleotide sequence of the paternal-specific allele at paternal-only heterozygous sites and determine the maternal transition by observing allelic imbalance at individual sites. However, the application of this simple idea could be hinderedby low cff-DNA concentration and sequence depth. In our plasma sequencing, approximately 57.84% of the paternal-specific alleles were totally absent (Figure S3). Additionally, our estimation of the concentrations of three different alleles in plasma showed that 24,938 of 137,567 (25.40%) sites showed an opposite allelic imbalance (Additional file 1, Supplementary Materials). These results indicate the infeasibility of SBSS in samples with low cff-DNA concentration and sequence depth. Thus, we introduced a sensitive HMM to identify the parentally transmitted allele and recombination breakpoints (Figure 2 and Additional file 1, Supplementary Methods), in which we predicted the fetal haplotype on the paternal-only, maternal-only, and biparentally heterozygous sites in one step. With the use of the HMM and Viterbi algorithm, the fetal haplotypes of the 374,980 markers (including chromosome X) were recovered successfully (Table 2).

**Table 2:** The general accuracy of haplotype prediction

Category		Paternal allele	Maternal allele	
		
		Autosome	Autosome	ChrX
**Consistent with g-DNA from cord blood**	Loci (*n*, percentage)	105,729 (98.57%)	103,082 (95.37%)	1,902 (98.45%)

**Inconsistent with g-DNA from cord blood**	Loci (*n*, percentage)	1,529 (1.43%)	5,005 (4.63%)	30 (1.55%)
	Type I (noisy from haplotype inference)	1,458 (95.36%)	1,442 (28.81%)	-
	Type II (recombination breakpoint related)	71 (4.64%)	3,295 (65.83%)	24 (80.00%)
	Type III (centromere or chromosome edge related)	0 (0%)	268 (5.35%)	6 (20.00%)

**Total**		107,258	108,087	1,932

### Accuracy of the recovered fetal haplotype

We performed a final validation to estimate the overall accuracy of the predicted fetal haplotype. To assess the standard fetal haplotype, we also performed a whole genome sequencing of the cord blood obtained after the child's birth to approximately 20-fold coverage (Table 1 and Additional file 1, Table S1). The genotypes of the child were determined using SOAPsnp and were validated at 99.25% consistency with his genotyping of HumanOmni2.5-8 BeadChip. The standard haplotype of the child was inferred by the same method as used for his parents. Finally, the general accuracy of the paternal alleles and maternal alleles were estimated by comparing the recovered fetal genome with the standard haplotype of the child (Table 2). For the recovered paternal alleles, 105,729 loci of our recovery were consistent with the standard haplotype, indicating a high accuracy of 98.57%. For the recovered maternal autosomal alleles, 103,082 loci were consistent with the standard haplotype, for a slightly lower accuracy of maternal allele recovery of 95.37%. The maternal allele recovery on the chromosome X showed an accuracy of 98.45% (1,902/1,932 loci).

We further classified the recovery errors into different types (Table 2). Type I errors, which were randomly distributed throughout the whole genome, explained 95.36% and 28.81% of paternal and maternal recovery inaccuracies, respectively. We assume that type I errors were caused by the haplotype ambiguity during the parental or standard fetal haplotype inference. The type II errors, which mostly clustered next to the recovered recombination breakpoints, were most probably caused by the inaccuracy of recombination breakpoint recovery. This type of error explained the remaining 4.64% of the paternal allele recovery inaccuracies, 65.83% of the maternal autosome allele recovery mistakes, and 80.00% of chromosome X recovery mistakes, indicating difficulties in maternal recombination breakpoint determination. The rest, referred to as type III errors, were related to heterochromatin close to the centromeres or chromosome ends. Type III errors explained 5.35% of maternal autosome and 20.00% of chromosome X maternal allele recovery errors (Additional file 1, Table S5).

**Figure 3:**
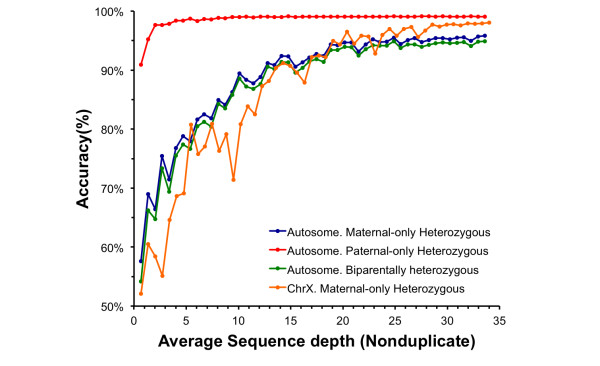
**The relationship between accuracy and sequence depth**. The color-coded curves denote statistics at different kinds of sites (blue: autosome, maternal-only heterozygous sites; red: autosome, paternal-only heterozygous sites; green: autosome, biparentally heterozygous sites; orange: ChrX, maternal heterozygous sites).

To estimate the correlation between sequencing data and the detection accuracy of the recovered fetal genome, we sampled a subset of data from the maternal plasma sequencing (Figure 3). Generally, the accuracy of the recovered fetal genome increased with the depth of maternal plasma sequencing. Because of the existence of type I errors, the accuracy began to stabilize when the sequence depth grew >20X. Additionally, the accuracy of the paternal-only heterozygous sites indicated the robustness of our method for paternal allele recovery in low cff-DNA concentrations among different sequence depths. For example, using only 6% of the plasma sequence data (non-duplicate approximately 2.01X), we successfully recovered 97.61% of the maternal-only heterozygous sites.

In summary, 98.57% of the paternal alleles, 95.37% of the maternal autosomes and 98.45% of the chromosome X were recovered precisely using approximately 43.91X (non-duplicate approximately 33.60X) maternal plasma sequencing with a 5.69% cff-DNA concentration. The quality of haplotype interference, the accuracy of the recombination breakpoint prediction, and heterochromatin close to centromeres and chromosome ends explained most of our recovery errors. The simulations suggested the robustness of our method at lower sequence depth and low cff-DNA concentration, especially for paternal allele recovery.

### The application of non-invasive fetal genomics

Inheritable genetic disease screening and Mendelian character predictions are two important applications of accurate fetal genome recovery. So far, 7,895 pathogenic genes related to Mendelian diseases have been released by OMIM (Online Mendelian Inheritance in Man [22]), 96.65% of which were directly covered by or strongly linked with our 374,980 effective marker loci. In the case of complex diseases, 98.90% of 6,939 disease-associated loci from the NHGRI GWAS Catalog [23] were directly covered by or strongly linked with our marker loci. Interestingly, a TC genotype at rs17822931 (Chr16: 46,815,699; predicted accurately) is consistent with the offspring having earwax of the wet type [24], which is not typical in Asians and Native Americans [25]. The level of throughput of disease and trait screening implies that noninvasive prenatal diagnosis/screening can have high detection efficiency. The strategy of using three generations of a family increased the robustness of detecting rare mutations, showing a similar performance between common mutations and rare mutations (Additional file 1, Figure S4). Moreover, based on our accurate recovered haplotype, heritable complex variations (such as long insertions/deletions, translocations, rearrangements, and even disease-related methylation), unlike SNPs, which were hard to observe directly in maternal plasma, could be mapped to the fetal genome by their linkage disequilibrium relationships using other existing techniques.

## Discussion

Here we report a haplotype-assisted approach for non-invasive fetal whole genome recovery. The characteristics of our method and two previously reported haplotype-assisted methods [15,16] are summarized in Table 3. We further assessed the fetal genotype accuracy to compare the practical performance in corresponding cases to reach comprehensive conclusions (Table 4).

**Table 3:** Comparison of fetal genome recovery methods

Category	Current study	**Kitzman*et al***.	Fan *et al*.
**Parental haplotype inference**			
Method for parental haplotype construction	Trio strategy with corresponding grandparents and CHS	Maternal: fosmid-based approach [26] Paternal: could not be assessed due to lowmolecular weight of saliva DNA	Maternal: single-cell approach [27] Paternal: not collected
**Strategy for fetal genome recovery**			
Paternalallele	Two different alleles of fetal haplotype, transmitted from the two parents, were reconstructed by a HMM model in one step, including transmitted chromosomes and recombination breakpoints	SBSS	SBSS+ imputation
Maternal allele		For maternal-only heterozygous sites, they used AIEto determine whole-block transitions and HMM to identify assembly errors and recombination breakpoints. For biparentally heterozygous sites, maternal alleles were determined by maternal-only heterozygous sites within the same block	Allele imbalance estimated by counting nucleotides specific to each of the two maternal alleles
**Recovery of fetal genome**			
Genotype	Yes	Yes	Yes
Haplotype	Yes	No	No
*De-novo *mutation	No	Yes	No

**Table 4:** Practical performance comparison between fetal genome recovery methods

Category		Current study	Kitzman *et al*.		Fan *et al*.		
			
			Trio I1	Trio G1	P1T1	P1T2	P2T3
Gestational week		13	18.5	8.14	9	29	39
Estimated average cff-DNA concentration		5.69%	13%^a^	6%^a^	6%^a^	16%^a^	30%^a^
Average sequence depth (fold)		43.91	78^a,b^	56^a,b^	52.7	20.8	10.7
Fetal gender		Male	Male	-	Female	Female	Female
**Maternal allele **							
Predicted rate		100%	91.4%	-	>99.2%		
Predictionaccuracy		95.37% (autosome) 98.45% (ChrX)	-	-	>99.8%		
**Paternal allele**							
Predicted rate		100%	-	-	71.60%	72.84%	72.94%
Prediction accuracy		98.57%	-	-	93.79%	95.84%	96.56%
**Accuracy of inferred fetal genotype**							
Autosome	Paternal-only heterozygous	99.12%, *n *= 65,409	96.8%	60.3%	-	-	-
	Maternal-only heterozygous	95.84%, *n *= 66,238	99.3%^c^	95.7%^c^	-	-	-
	Biparentally heterozygous	94.90%, *n *= 41,849	98.7%^d^	91.3%^d^	-	-	-
ChrX	Maternal-only heterozygous	98.45%, *n *= 1,932	-	-	-	-	-

- = No data

^a^Approximate.

^b^Non-duplicate.

^c^Estimated based on maternal phased sites.

^d^Accuracy of transmitted maternal allele prediction, estimated based on maternal phased sites.

Three fetal genome recovery strategies have employed parental haplotypes, but with different inference strategies. We used a common genetics approach to determine the parental haplotypes by using the genotyping data of surviving grandparents or born offspring. This approach provided a practical strategy for non-invasive fetal genome screening for families with probands, especially for families with born offspring with Mendelian diseases. However, this specific sample recruitment would restrict its prospects for clinical application. To overcome this limitation, Kitzman *et al. *[26] and Fan *et al. *[27] have constructed maternal haplotypes directly using noninvasive experimental approaches. However, the time and resource consumption of their complex experimental methods would restrict their clinical application. For example, it would take approximately 8 days and another US$3,678 to prepare the fosmid clone library [26].

Second, these three strategies all used the maternal haplotype, but we used the paternal haplotype. For paternal-only heterozygous sites, Kitzman *et al. *performed SBSS to detect the paternal-specific allele. In SBSS, one or more reads matching the paternal-specific allele are taken as evidence of its transition. However, the performance of SBSS depends on cff-DNA concentration and sequence depth. For example, 96.80% of alleles in the trio labeled I1 in the Kitzman *et al. *study (WGS approximately 78X, cff-DNA concentration approximately 13%) were predicted correctly. However, in the case of low cff-DNA concentration, such as trio G1 (WGS approximately 56X, at 8.14 weeks) reported by Kitzman *et al*., only 60.3% of paternal-specific alleles were identified correctly. Therefore, Fan *et al. *imputed the paternal allele using data from the 1000 Genomes project. In total, approximately 70% of the paternally transmitted alleles were reconstructed with an accuracy of 93% to 97%. This implied the imperfect efficiency and eurytopicity of SBSS, even with population-scale sequencing for imputation. The dependence on cff-DNA concentration and sequence depth also limits the application of SBSS to early gestation. Therefore, it is an advisable strategy to use the paternal haplotype for noninvasive fetal whole genome recovery, especially for Mendelian disease diagnosis.

Third, the results of the fetal genome recovery were different between the three studies. Kitzman *et al. *and Fan *et al. *focused on fetal genotype inference. We tried to recover the fetal haplotype and genotype because the haplotype information is important for complex diseases screening, such as systemic lupus erythematosus [18]. Besides helping in haplotype-related disease detection, accurate fetal haplotype prediction might be helpful to identify fetal *de-novo *copy number variations, such as aneuploidy or even microdeletion and microduplication syndromes (Additional file 1, Supplementary Materials).

Fourth, only Kitzman *et al. *performed a fetal *de-novo *mutation identification. These mutations were expected to appear within the maternal plasma as rare alleles, like paternal-specific alleles [15]. Ideally, SBSS can identify the fetal *de-novo *mutations easily. Therefore, Kitzman *et al. *achieved 88.60% sensitivity of high-confidence fetal *de-novo *mutations. However, the systematic error, which was dominated by errors originating during polymerase chain reaction (PCR), introduced hundreds of noisy signals. Even with stringent filters, >99% of the candidate sites were false-positive, implying a specificity of approximately 0.84% in trio I1. Moreover, as mentioned above, the sensitivity of SBSS depends on cff-DNA concentration and sequence depth. In our case, only 68.97% of the high-confidence fetal *de-novo *mutations could be identified (Additional file 1, Table S4). Hence, effective algorithms are still required for fetal *de-novo *mutation identification.

In conclusion, all three of these methods provide promising solutions for non-invasive fetal genome recovery, with different strengths and weaknesses. The performance of paternal allele recovery indicated the requirement of the paternal haplotype, especially for non-invasive Mendelian disease detection. Therefore, it is wise to choose a suitable approach to obtain the parental haplotype based on the clinical reality, and our data show that a strategy with additional relative samples would be an alternative method. Our strategy of parental haplotype inference provided a practical solution to detect fetal Mendelian diseases non-invasively, especially for couples with a born proband.

Currently, it costs US$41 to generate a single gigabyte of sequence data with the Illumina HiSeq 2000 platform [28]; therefore, it would cost at least US$14,000 to generate the sequence data in this study. With developing technology, the price of the sequencing will drop to US$1,000 per genome in the foreseeable future [29]. Consequently, sequence-based approaches will become practical for non-invasive fetal whole genome recovery. Regarding Mendelian diseases, combining our method with exome sequencing technology, it will cost only US$1,200-1,400 for each family with a born proband (including 30X exome sequence coverage for the couple, the born proband, and the maternal plasma), which is affordable for many families. Moreover, the developing sequence platforms with shorter turnaround times will significantly broaden the application of sequence-based approaches for fetal genome recovery. For example, the MiSeq platform takes <48 h for PE 150 sequencing, meaning pregnant women could receive their results within 1 week. Thus, the advantage of short turnaround times makes us confident that sequence-based approaches for fetal genome recovery will play an increasingly important role in the future.

The development of non-invasive measures for fetal genome recovery will surely bring new insight to prenatal genetic diagnosis. In the case of fetal Mendelian disease identification, sequence-based approaches will provide fast and reliable options to pregnant women, reducing the use of unnecessary invasive procedures. Comprehensive fetal genome sequencing with high accuracy not only enables us to make definitive diagnoses but also provides potential applications in personal medicine, such as identification of allergens [30]. In addition, the easy sampling of sequence-based approaches shows eurytopicity for gestational stage, which may be helpful to make appropriate clinical decisions. For instance, a fetus diagnosed with phenylketonuria in the third trimester would benefit from treatment immediately after delivery [16]. However, the increase in information available to parents will raise ethical questions. For example, in most cases, the influence of a novel fetal mutation is hard to predict. Should a woman be informed if her fetus has a novel mutation of unpredictable consequence? The uncertainty of these mutations may increase the unnecessary anxiety of pregnant women; however, the lack of such information would lead to improper decisions. Thus, the key concern is what kind of information would/should be reported, and this question should be thoroughly discussed within the scientific community and on a societal level.

There are still several limitations of our approach hampering further clinical application. First, the use of commercial microarrays (grandparents or the CHS trios) greatly restricted our study of common SNPs. Therefore, only a small fraction of SNPs were discussed in our study, and we ignored most of the rare variations. In addition, short indels, which could not be located in the parental haplotypes because of the lack of grandparental information, were excluded from our analysis. Short indels not only play an important role in Mendelian disease [31] but also show strong power as markers [32]. At present, target sequences with abundant tag-SNPs are advisable for future studies. Second, our study focused on mutations at the DNA level, which excluded most of the haplotype-associated transgenerational epigenetic modifications [33]. The clinical application of fetal genome recovery will require more robust experimental breakthroughs and algorithms to explore the comprehensiveness of the genome coverage. Third, although we successfully recovered a fetal genome in a case of a singleton pregnancy, accurate genome recovery in cases of twin pregnancy is still unattainable. Currently, the sequence-based approach for non-invasive prenatal diagnosis in twin pregnancies is restricted to aneuploidy [34]. The recovery of twins' genomes will greatly broaden the horizon of non-invasive prenatal diagnosis.

## Conclusions

In this study, we introduced an accurate method for fetal genome recovery in one step using maternal plasma sequencing. More than 95% of the fetal genotypes were inferred successfully, and most importantly, >95% of the fetal haplotypes were recovered precisely. As a proof of concept, we propose the clinical application of the recovered genome to non-invasive prenatal diagnosis/screening. In summary, we report an accurate and easy method for non-invasive fetal whole genome recovery by maternal plasma sequencing. An accurate fetal haplotype would enhance the dimensionality of fetal variation detection in prenatal diagnosis/screening and promote the development of fetal medicine. Our results indicate the potential of using sequencing technology in prenatal diagnosis, and they should accelerate the application of sequencing technology in clinical trials.

## Abbreviations

cff-DNA: cell-free fetal DNA; g-DNA: genomic DNA; HMM: hidden Markov model.

## Competing interests

The authors have read the journal's policy and have the following competing interests to declare: Shengpei Chen, Huijuan Ge, Xuchao Li, Jian Wang, Jun Wang, Huanming Yang, and Xiuqing Zhang have filed patent applications on non-invasive fetal genome recovery by massively parallel sequencing of maternal plasma. The patent number is PCT/CN2012/075478 (date 2012-5-14). The remaining authors declare that they have no competing interests.

## Authors' contributions

XZ, YL, HY, JiW, JuW, SL, and WW managed the project. YL, XZ, and SC designed the analysis principle. SC, XW, XL, XY, CZ, HZ, and FJ performed the data analysis. HG, XP, FC, HJ, PL, LeZ, and LiZ performed the sequencing. SC, HG, and XP prepared this manuscript. All authors read and approved the final manuscript.

## Supplementary Material

Additional file 1 **Supplementary materials**. This file provides a detailed description of the experimental work and bioinformatics methods of this study (SNP calling, parental haplotype construction, fetal genome recovery). We also performed comprehensive maternal plasma DNA profiling (section 2). This file further contains supplementary Figures S1-5 and Tables S1-3: Figure S1, 'The size distribution of plasma DNA', shows the size distribution of maternal and fetal DNA fragments in DNA. Figure S2, 'The GC content of plasma DNA segments', shows the GC consistency between maternal and paternal DNA segments in plasma. Figure S3, The sequence depth of paternal-specific alleles', shows the sequence depth distribution of paternal-specific alleles in maternal plasma. Figure S4, 'The distribution of the concentration difference between allele 0 and allele 1', shows the concentration difference between the maternal alleles in plasma. Table S1, 'The consistency between SNP calling using NGS data and the Illumina 2.5M array', shows the SNP calling consistency between NGS and the SNP-array. Table S2, 'The average of estimated concentration of each allele on different chromosomes', shows the average concentration of each allele on different chromosomes calculated by MLE. Table S3, 'The cff-DNA concentration of each chromosome based on paternal-specific alleles', shows the average cff-DNA concentration on each chromosome. Table S4, 'The read distribution of fetal *de-novo *mutations', shows the distribution of reads in plasma sequences at high-confidence fetal *de-novo *mutation sites. Table S5, 'The type III errors', lists detailed information of the type III errors of fetal genome recovery. Click here for file
