# Supplementary material for: Haplotype-assisted accurate non-invasive fetal whole genome recovery through maternal plasma sequencing
Source: Genome Med. 2013 Feb 27;5(2):18. doi: 10.1186/gm422 (PMC3706925; doi:10.1186/gm422)
Supplement: Additional file 1 — Supplementary materials. This file provides a detailed description of the experimental work and bioinformatics methods of this study (SNP calling, parental haplotype construction, fetal genome recovery). We also performed comprehensive maternal plasma DNA profiling (section 2). This file further contains supplementary Figures S1-5 and Tables S1-3: Figure S1, 'The size distribution of plasma DNA', shows the size distribution of maternal and fetal DNA fragments in DNA. Figure S2, 'The GC content of plasma DNA segments', shows the GC consistency between maternal and paternal DNA segments in plasma. Figure S3, The sequence depth of paternal-specific alleles', shows the sequence depth distribution of paternal-specific alleles in maternal plasma. Figure S4, 'The distribution of the concentration difference between allele 0 and allele 1', shows the concentration difference between the maternal alleles in plasma. Table S1, 'The consistency between SNP calling using NGS data and the Illumina 2.5M array', shows the SNP calling consistency between NGS and the SNP-array. Table S2, 'The average of estimated concentration of each allele on different chromosomes', shows the average concentration of each allele on different chromosomes calculated by MLE. Table S3, 'The cff-DNA concentration of each chromosome based on paternal-specific alleles', shows the average cff-DNA concentration on each chromosome. Table S4, 'The read distribution of fetal de-novo mutations', shows the distribution of reads in plasma sequences at high-confidence fetal de-novo mutation sites. Table S5, 'The type III errors', lists detailed information of the type III errors of fetal genome recovery. [file gm422-S1.PDF]

Supplementary

Haplotype-assisted accurate non-invasive fetal whole genome recovery through maternal plasma sequencing

Shengpei Chen[1, 2†], Huijuan Ge[1†], Xuebin Wang[1†], Xiaoyu Pan[1, 3†], Xiaotian Yao[1], Xuchao Li[1], Chunlei Zhang[1], Fang Chen[1], Fuman Jiang[1], Peipei Li[1], Hui Jiang[1], Hancheng Zheng[1], Lei Zhang[1], Lijian Zhao[1], Wei Wang[1], Songgang Li[1], Jun Wang[1], Jian Wang[1], Huanming Yang[1], Yingrui Li[1]*, Xiuqing Zhang[1]*

[1] BGI-Shenzhen, Shenzhen 518083, China

[2] State Key Laboratory of Bioelectronics, School of Biological Science and Medical Engineering, Southeast University, Nanjing 210096, China

[3] School of Bioscience and Bioengineering, South China University of Technology, Guangzhou 510000, China

[†] These authors contributed equally to this work.

* Correspondence: Yingrui Li; Xiuqing Zhang. Email: liyr@genomics.org.cn; zhangxq@genomics.org.cn

Contents

Bioinformatics **3**

Short reads alignment and parental SNP calling 3

Parental haplotype inference 3

Fetal haplotype recovery with HMM and Viterbi algorithm 3

Offspring's SNP calling and standard haplotype inference 4

Maximum likelihood estimation Cff-DNA concentration 5

Chromosomal abnormalities and micro deletion/duplication idenfication using recovered haplotype 6

Comprehensive plasma DNA profiling **6**

Supplementary figures **8**

Figure S1. The size distribution of plasma DNA 8

Figure S2. The GC content of plasma DNA segments 9
Figure S3. The sequence depth of paternal specific allele 10
Figure S4. The distribution of the concentration difference between allele 0 and allele 1 11
Supplementary tables 12
Table S1. The consistency between SNP calling using NGS data and Illumina 2.5M array 12
Table S2. The average of estimated concentration of each allele on different chromosomes 13
Table S3. The cff-DNA concentration of each chromosome based on paternal specific allele 14
Table S4. The reads distribution on fetal *de-novo* mutation 15
Table S5. Type III errors 16
Acknowledgements 17
References 17

Bioinformatics

Short reads alignment and parental SNP calling

Short reads generated by Illumina Hiseq 2000 were mapped to the human reference (NCBI 36) using SOAP2 [1]. Afterwards, we performed SNP calling using SOAPsnp [2] with default parameters. And we applied filters (Q >20 and depth >=4) to guarantee the accuracy of the parental and standard fetal genotype. Thus, our genotype showed a high consistency of approximately 99.22% with the SNP array (Table 1 and Supplementary Table S1).

Parental haplotype inference

We constructed the parental haplotype using a combination strategy of trio and unrelated individuals. For autosome, the parental haplotype were inferred by the genotype information of grandparents with the newly released 51 parent-offspring trios of Chinese Han in 1000 genome project using BEAGLE [3] (default parameters). For chromosome X, because of the haploidy and absence of recombination of males, mother's haplotype could be easily inferred with maternal grandfather's genotype on chromosome X.

Fetal haplotype recovery with HMM and Viterbi algorithm

1. Basic denotation

The number of the loci on certain chromosome was denoted as N_c while the total number of the all loci was denoted as N^*. And the parental haplotypes were recorded as $FH = \{fh_0, fh_1\}$ and $MH = \{mh_0, mh_1\}$, where $mh_k = \{m_{i,k}\}$, $fh_k = \{f_{ik}\}$, $k \in \{0,1\}$, $i = 1,2, 3.., N_c$ and $\forall fh_{i,k}, m_{i,k} \in \{A, C, G, T\}$. For the unknown fetal haplotype, we noted it as $H = \{h_0, h_1\}$ where $h_0 = m\{i,x_i\}$ and $h_1 = f\{i,x_i\}$. Therefore, $q_i = \{x_i, y_i\}$ consists of the hidden state that we need to decipher, and all potential hidden states consist of the set Q.

In maternal plasma sequencing, we denoted sequence base as $S = \{S_i\}$, where $S_i = \{n_{i,A}, n_{i,C}, n_{i,G}, n_{i,T}\}$ standing for the sequence depth of each base.

For other parameters in maternal plasma, the average cff-DNA concentration and the average sequence error was denoted as ε and e.

2. Initial state distribution $\pi = \{\pi_j\}, j \in Q$. Since the lack of prior probability, we defined $\pi_j = \Pr(q_1 = j)$ [1-4], representing the same initial probability of each hidden state.

3. Transition probabilities matrix $A = \{a_{jk}\}$ $(j, k \in Q)$, where $(1-p_r)$ $x_i = x_{i\text{-}1}, y_i = y_{i\text{-}1}$ $a_{jk} = \Pr$ $(q_i = k\ q_{i\text{-}1} = j) = (1\text{-}p_r) \cdot p_r\ x_i = x_{i\text{-}1}, y_i \neq y_{i\text{-}1}\ or\ x_i \neq x_{i\text{-}1}, y_i = y_{i\text{-}1}\ p^2\ x \neq x\ \text{-}\ , y \neq y\ \text{-}\ r.$ And $p_r = re$ N^*, re was the average frequency of the recombination between gemmate, where we used $re = 30$ in our case.

4. Observation symbol probabilities matrix $B = \{b_{i,j}(s_i)\}$ $(j \in Q)$, where $b_{i,j}(s_i) = \Pr(s_i\ q_i$ $= j, \{m_0, m_1\}) = ({}^n i, A + {}^n i, C + {}^n i, G + {}^n i, T)! \cdot (P_{i,A})^n i, A \cdot (P_{i,C})^n i, C \cdot (P_{i,G})^n i, G \cdot (P_{i,T})^n i, T^n i,$ $A\ {}^{!n} i, C\ {}^{!n} i, G\ {}^{!n} i, T!$

And $P_{i,base} = \Pr(base\ q_i = j, \{m_0, m_1\}) = \sum 1\ (1\text{-}\varepsilon)\ (base, m_k) + 1\ \varepsilon \cdot \Delta\ (base, m_{xi}) + 1\text{-}\ \varepsilon \cdot \Delta$ $(base, f_{yi})\ k \in \{0,1\}$ [2]. And the indicator function $(x, y) = {}^1\text{-}\ {}^e\ x = y\ e\ 3\ x \neq y$

5. Viterbi algorithm [4]

(1) Initialization $\delta_1(q_1) = \pi_j \cdot b_{1q1}(s_1)$

(2) Iteration $\max \delta\text{-}(q\text{-}) \cdot a\ {}^{\delta}i\ ({}^q i) = ({}_{qi\text{-}1 \in Q}\ {}^{I\,1\,q} i\text{-}1^q i)\ {}^b i, q_i(s_i), \Psi_i(q_i) = \text{argmax}\, \delta_{i\text{-}1}(q_{i\text{-}1}) \cdot a_{qi\text{-}1qi}\ {}^q i$ $-1^{\in Q}$

(3) Termination and back tracking

The final optimized hidden state $* = \delta q\ {}^q N_c\ \text{argmax}\ N_c\ ({}^N c)\ {}^q N_c\ {}^{\in Q} * (q_i)$. And the optimized path ${}^q i\text{-}1\ {}^{-\Psi} i\ i = 2, 3, \ldots, N_c$

Offspring's SNP calling and standard haplotype inference

The short reads generated by cord blood were mapped to the human reference genome and received SNP calling with same pipeline with his parents. The fetal standard haplotype were inferred with his parents and 51CHS parent-offspring trios using BEAGLE. For chromosome X, because of the haploidy of the male offspring, we directly obtained his haplotype using SOAPsnp.

Maximum likelihood estimation Cff-DNA concentration

The concentration of certain allele was defined as relative percentage comparing to all allele. And we denoted the concentration of each allele as

$$\varepsilon_j = \frac{\text{the DNA amount of allele}_j}{\sum_{j=0}^{2} (\text{the DNA amount of allele}_j)}$$

where subscript j denoted the allele id (0 for maternal allele that passed to offspring; 1 for maternal allele that did NOT pass to offspring; 2 for paternal allele that passed to offspring). Then we could classify all sites through the heterozygosity of mother and fetus into four categories, and we denoted the expected probability of each base in maternal plasma sequencing as following table.

	Heterozygosity		Sequence base	
Type	Mother	Offspring	Same as allele 0	Different from allele 0
L1	Homo.	Homo.	-	-
L2	Heter.	Homo.	$\varepsilon_0 + \varepsilon_2$	ε_1
L3	Homo.	Heter.	$\varepsilon_0 + \varepsilon_1$	ε_2
L4	Heter.	Heter.	ε_0	$\varepsilon_1 + \varepsilon_2$

And the corresponding sequence depth of base same with or different from allele0 were noted as $d_{i,0}$ and $d_{i,1}$ with subscript i as loci id. The likelihood probabilities could be evaluated as, $d_{i,0}\ d_{i,1}\ d_{i,0}\ d_{i,1}\ d_{i,0}\ d_{i,1}\ L = \prod C_i\ (\varepsilon_0 + \varepsilon_2)\ (\varepsilon_1) \cdot \prod C_i\ (\varepsilon_0 + \varepsilon_1)\ (\varepsilon_2) \cdot \prod C_i\ (\varepsilon_0)\ (\sum 1 + \sum 2)\ L_2\ L_3\ L_4$ where C_i was the Bernoulli coefficient. With regard to $\varepsilon_0 + \varepsilon_1 + \varepsilon_2 = 1$, we could solve the optimized concentration using function *optim* in R [5].

Chromosomal abnormalities and micro deletion/duplication idenfication

using recovered haplotype

(1) Based on recovered fetal haplotype, calculated the average concentration of each loci using the MLE method with extend K-bp region. (Denote as $\varepsilon_{0,i}$, $\varepsilon_{1,i}$, and $\varepsilon_{2,i}$; subscript i for different sites).

(2) Define the statistics concentration balance, $b_i = \varepsilon_{2,i} \varepsilon_{0,i} - \varepsilon_{1,i}$.

(3) Merge sites into segments using optimized iteration [6].

a. Initialization. In this section, we would initialize candidate copy number variation breakpoints. For each sites, we defined left local bins that contained *n* loci, and right local bins in the same way. In the other words, $bin_{left,i} = \{b_{i-n}, ..., b_i\}$ and $bin_{right,i} = \{b_i, ..., b_{i+n}\}$. We then recruited Run test to examine the concentration balance difference between the left and right local bins, and the top a_{ini} sites with the most significant *P* values would be considered as candidate copy number variation breakpoints ($CB = \{i_0, i_1, \ldots, i_{a_{ini}}\}$).

b. Iteration. In each round, the least significant candidate breakpoint would be removed until only a_{final} breakpoints left. For each candidate breakpoint, its significance was described by *P* value of the run test between left ($Segment_{left,ik} = \{b_{i_{k-1}}, ..., b_{i_k}\}$) and right ($Segment_{right,ik} = \{b_{i_k}, ..., b_{i_{k+1}}\}$) segments.

(4) Calculate the average concentration and balance of each segment.

(5) Segments with abnormal concentration balance would be regard as candidate chromosomal abnormalities or micro-deletion/duplication syndrome signals.

Comprehensive plasma DNA profiling

To explore the potential and limitation of non-invasive diagnosis using maternal plasma, we perform a comprehensive analysis on cff-DNA. For linguistic simplicity, the alleles which providing DNA to maternal plasma were referred as alleles 0, 1, and 2. The numbers 0, 1, and 2 stood for the maternal allele passed to the offspring, the other maternal allele, and the paternal allele respectively. To access the size distribution of the cff-DNA, reads pairs mapped to maternal homozygous and fetal heterozygous were recruited. The size of the DNA segments belonging to different alleles was calculated (Supplementary Figure S1). The DNA from maternal alleles

are most abundant at 166 bp in length while the size of cff-DNA was more dispersive relatively with pins approximately 10 bp apart, which was claimed to cause by 10-bp periodicity reminiscent of nuclease-cleaved nucleosomes and the enzymatic processing of DNA from apoptotic cells [7]. In terms of GC content, the cff-DNA segments showed no significant difference between other plasma DNA (Supplementary Figure S2). In the analysis of the sequence depth of paternal allele, we found that approximately 74.82% of paternal specific allele could be covered less than twice in our case (Supplementary Figure S3).

At last, we performed maximum likelihood estimation (MLE) for the concentration of each allele for each locus (Supplementary Method). In total, we successfully estimated the concentrations on 137,567 sites. On average, the concentrations of these three alleles were 49.97%, 47.15%, and 2.87%, respectively (Supplementary Table S2). Therefore, the concentration of the cff-DNA would be approximately 5.70%, close to that estimated using paternal specific allele (5.67%, Supplementary Table S3); however, cff-DNA concentration estimated by chromosome Y (10.20%) showed a significant difference from these two estimations. Also, we found that the genome-wide concentration distribution of each allele indicated a complicate situation of the maternal background. In theory or at the macro level, the concentration of allele 0 in plasma should be larger than that of allele 1, since both mother and fetus provide cell free DNA for allele 0 while only mother for allele 1. However, 25.40% (24,938/137,567) sites showed an opposite situation (Figure 1c and Supplementary Figure S3), which would lead to a segmental recovery error such as the spread mistakes in Lo’s study [8]. This obstacle could be overcome by considering about the relationship between loci or blocks/segments, such as the transition probability referred before.

To sum up, the size and GC distribution of the cff-DNA in our study consisted with previously researches. The instability of the paternal allele distribution, and the complexity of the maternal background revealed by the MLE for the concentration of each allele, described the obstacles of the non-invasive diagnosis using maternal plasma sequence, and implied the robustness of our method to some extent.

Supplementary figures

Figure S1. The size distribution of plasma DNA. In this figure, we illustrated the size distribution of the five kinds of cell free DNA in maternal plasma. DNA from maternal alleles was label as dot line (light blue, autosome allele 0 and 1; purple, chrX allele 0; dark blue, chrX allele 1), and that from paternal specific allele was denoted as solid line (red, autosome allele 2; orange, chrY).

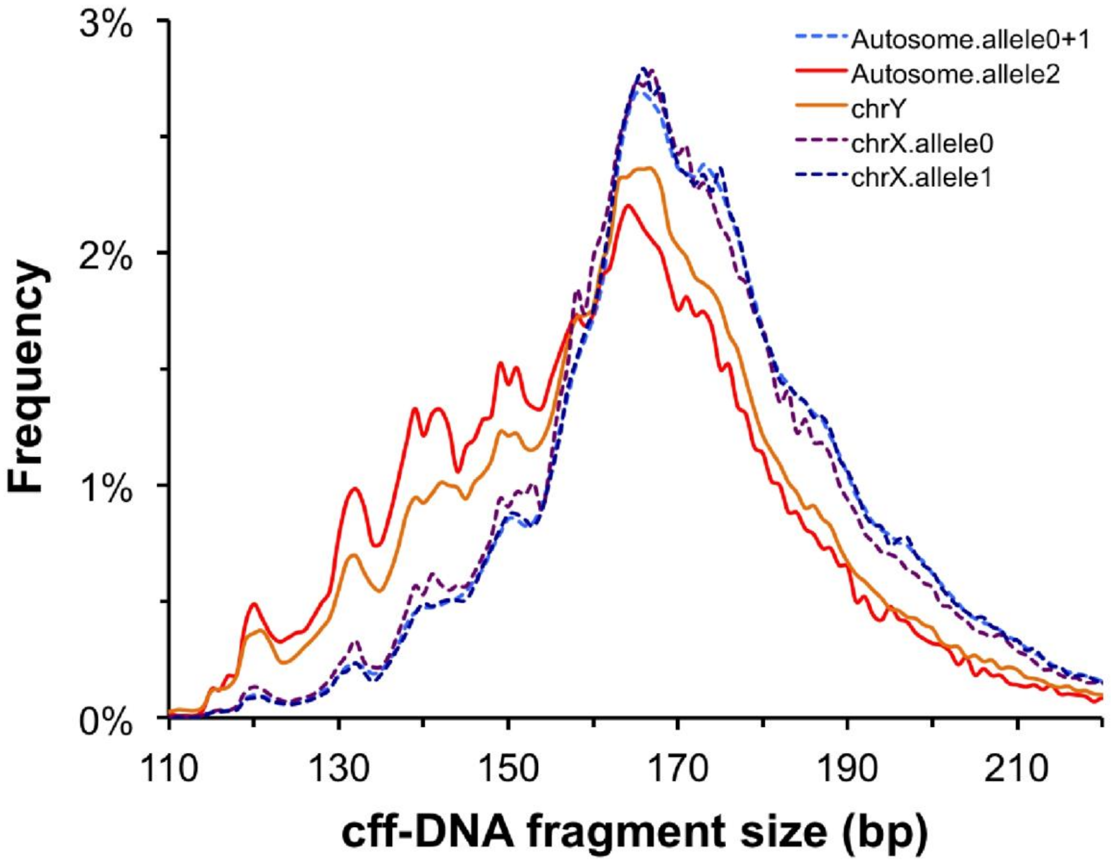

Figure S2. The GC content of plasma DNA segments. The GC content distributions of maternal background (blue dot line) and paternal specific allele (red solid line) were showed. In this figure, we found that there was no significant difference between these two curves.

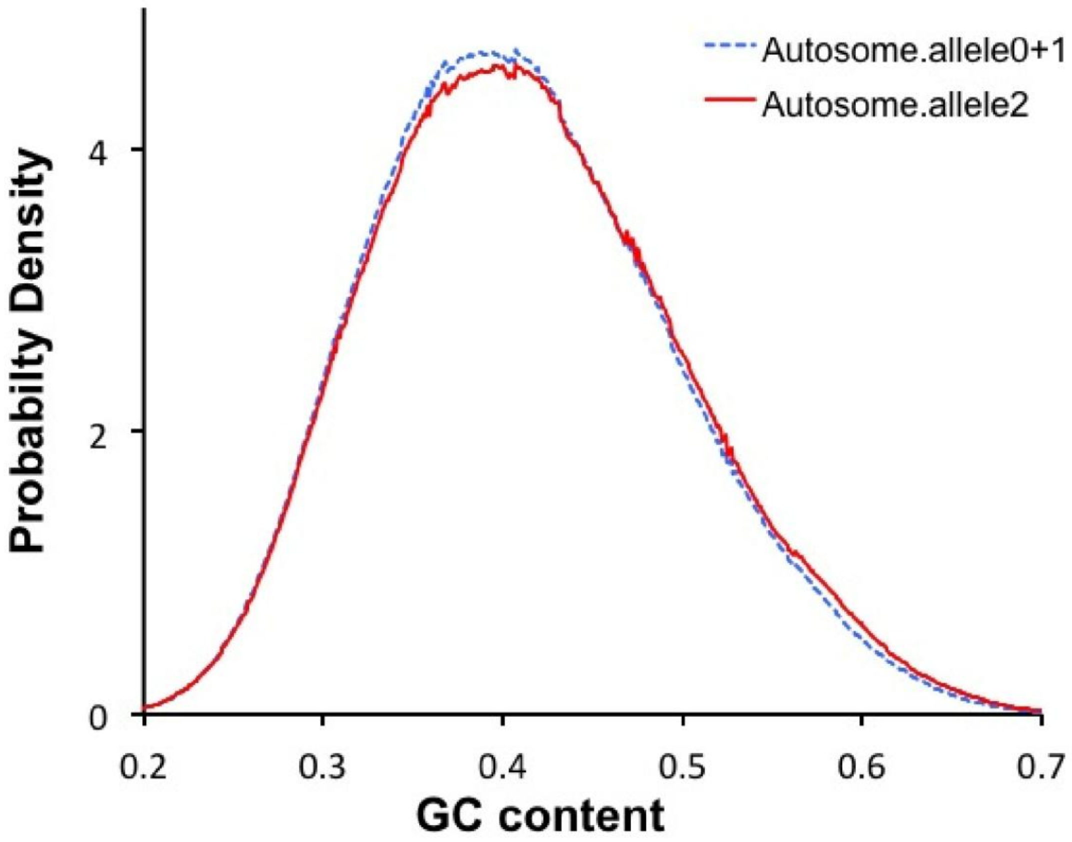

Figure S3. The sequence depth of paternal specific allele. In the maternal plasma, the sequence depth of paternal specific allele was usually used to estimate the cff-DNA concentration. However, 57.84% of the paternal specific allele was not covered in our study.

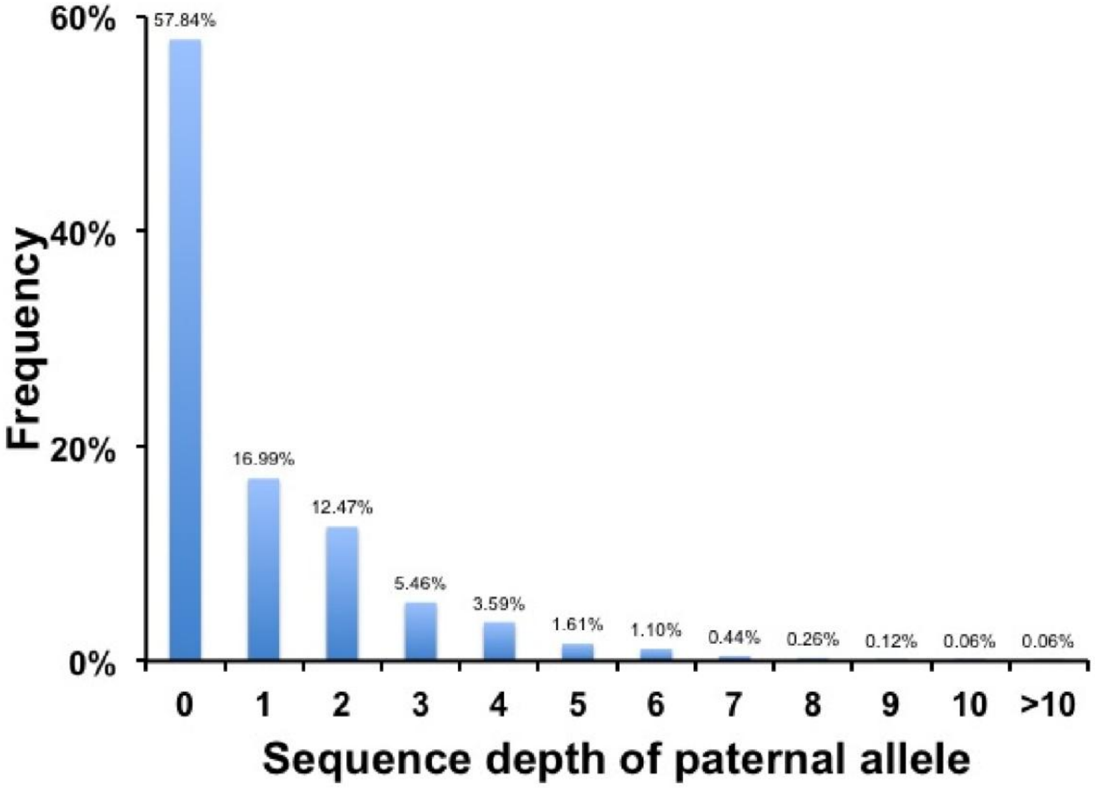

Figure S4. The distribution of the concentration difference between allele 0 and allele 1. To illustrate the concentration difference between alleles 0 and 1, we plotted the distribution of difference in this figure. Ideally, the concentration of allele 0 in plasma should be larger than that of allele 1, since both mother and fetus provide cell free DNA for allele 0 while only mother for allele 1; however, 25.40% (24,938/137,567) sites showed an opposite situation.

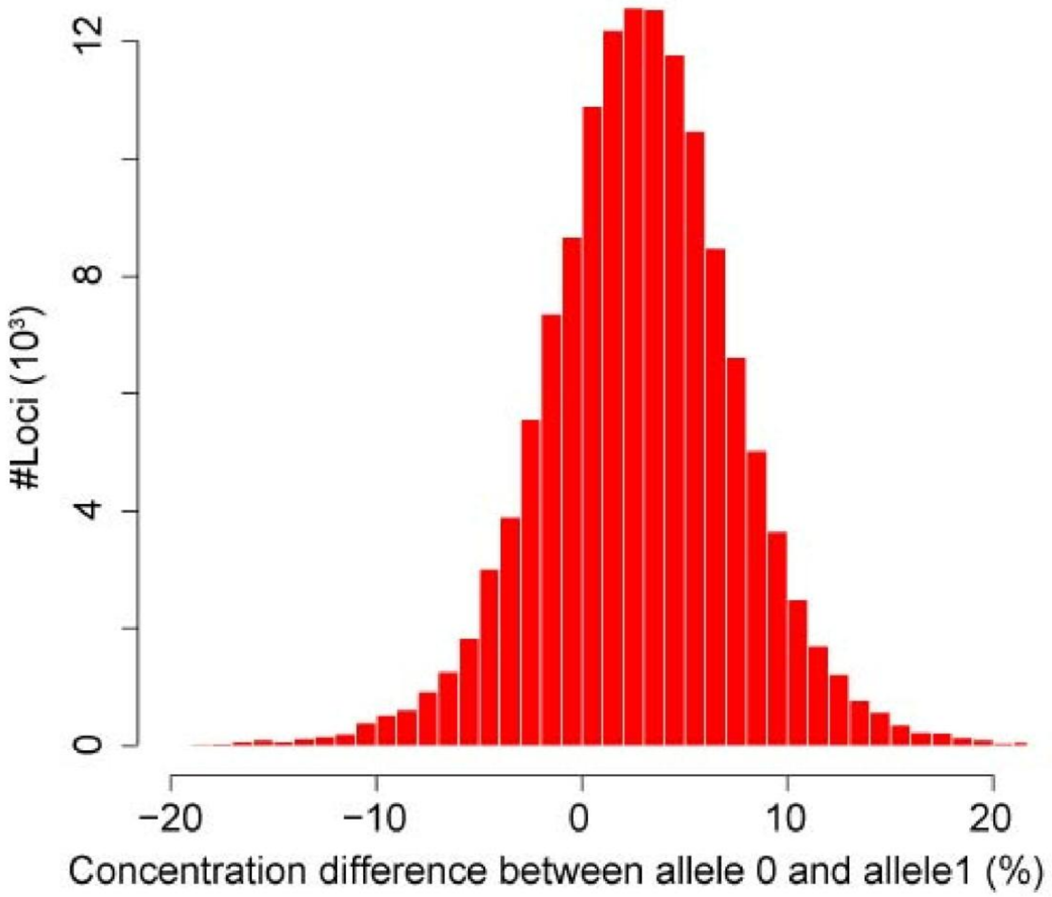

Supplementary tables

Table S1. The consistency between SNP calling using NGS data and Illumina 2.5M array

CHR	Father	Mother	Offspring
1	99.19%	99.20%	99.21%
2	99.05%	99.05%	99.11%
3	99.27%	99.24%	99.29%
4	99.20%	99.18%	99.21%
5	99.27%	99.21%	99.31%
6	98.95%	98.96%	99.04%
7	99.28%	99.25%	99.28%
8	99.33%	99.31%	99.34%
9	99.33%	99.25%	99.33%
10	99.29%	99.22%	99.24%
11	99.25%	99.17%	99.29%
12	99.29%	99.22%	99.30%
13	99.21%	99.13%	99.22%
14	99.33%	99.31%	99.33%
15	99.26%	99.29%	99.32%
16	99.29%	99.26%	99.30%
17	99.24%	99.17%	99.18%
18	99.19%	99.19%	99.25%
19	99.24%	99.23%	99.33%
20	99.32%	99.22%	99.34%
21	99.30%	99.18%	99.33%
22	99.31%	99.21%	99.35%
X	98.93%	99.37%	99.11%
Total	99.23%	99.19%	99.25%

Table S2. The average of estimated concentration of each allele on different chromosomes

CHR	Allele 0	Allele 1	Allele 2	Cff-DNA concentration
1	50.00%	47.23%	2.77%	5.55%
2	49.93%	47.29%	2.78%	5.43%
3	50.02%	47.11%	2.87%	5.78%
4	49.92%	47.15%	2.93%	5.71%
5	49.98%	47.14%	2.89%	5.73%
6	50.07%	47.08%	2.85%	5.84%
7	49.87%	47.24%	2.89%	5.53%
8	49.82%	47.39%	2.79%	5.23%
9	49.97%	47.19%	2.84%	5.62%
10	49.95%	47.21%	2.84%	5.57%
11	50.15%	47.01%	2.84%	5.97%
12	49.98%	47.25%	2.77%	5.50%
13	49.94%	47.19%	2.86%	5.61%
14	50.10%	47.02%	2.88%	5.96%
15	50.01%	47.14%	2.85%	5.72%
16	49.94%	47.24%	2.82%	5.52%
17	49.62%	47.57%	2.81%	4.86%
18	49.90%	47.24%	2.86%	5.53%
19	50.16%	46.79%	3.05%	6.42%
20	50.04%	46.96%	3.00%	6.09%
21	49.94%	46.95%	3.11%	6.09%
22	50.11%	46.93%	2.96%	6.14%
X	53.10%	46.90%	-	6.20%

Table S3. The cff-DNA concentration of each chromosome based on paternal specific allele

CHR	Estimated cff-DNA concentration
1	5.59%
2	5.42%
3	5.75%
4	5.93%
5	5.92%
6	5.81%
7	5.74%
8	5.44%
9	5.56%
10	5.64%
11	5.35%
12	5.68%
13	5.56%
14	5.96%
15	5.49%
16	5.74%
17	5.62%
18	5.74%
19	5.94%
20	6.07%
21	5.81%
22	5.39%
Total	5.67%

Table S4. The reads distribution on fetal *de-novo* mutation

Chr	Pos	Ref	Genotype of g-DNA			Reads depth of plasma				
			Father	Mother	Fetal	A	C	G	T	Total
1	146,618,643	G	GG	GG	CG	0	9	37	0	46
1	166,816,189	T	TT	TT	CT	0	0	0	40	40
2	175,156,552	T	TT	TT	AT	2	0	0	39	41
3	9,866,561	T	TT	TT	CT	0	3	0	36	39
3	9,866,562	G	GG	GG	AG	3	0	36	0	39
5	89,069,396	C	CC	CC	CT	0	60	0	3	63
6	89,183,193	C	CC	CC	CT	0	40	0	1	41
7	57,665,120	T	TT	TT	CT	0	5	0	81	86
7	61,426,329	A	AA	AA	AG	53	0	5	0	58
7	97,361,327	G	GG	GG	CG	0	1	53	0	54
8	146,271,567	T	TT	TT	CT	0	0	0	70	70
8	146,271,568	G	GG	GG	AG	0	0	64	0	64
9	67,851,109	C	CC	CC	CT	0	95	0	0	95
9	67,899,924	A	AA	AA	AG	41	0	7	0	48
9	68,359,443	G	GG	GG	GT	0	0	78	4	82
9	137,469,322	A	AA	AA	AG	49	0	4	0	53
10	6,909,546	A	AA	AA	AT	48	0	0	6	54
11	33,843,277	C	CC	CC	CT	0	48	0	2	50
11	59,971,410	T	TT	TT	AT	0	0	0	65	65
12	80,593,264	C	CC	CC	AC	0	36	0	0	36
14	18,517,398	C	CC	CC	CT	0	60	0	4	64
15	19,237,335	A	AA	AA	AT	59	0	0	3	62
15	19,237,336	G	GG	GG	CG	0	3	57	0	60
15	19,358,457	C	CC	CC	CT	0	79	0	3	82
18	15,160,924	G	GG	GG	CG	0	0	61	0	61
21	13,320,642	A	AA	AA	AG	35	0	2	0	37
22	15,319,333	T	TT	TT	AT	3	0	0	33	36
22	22,210,340	C	CC	CC	CT	0	42	0	0	42
22	22,210,343	G	GG	GG	AG	0	0	40	0	40

Table S5. Type III errors

CHR	Start	End	#Markers	Notes
1	114,536,765	120,982,136	179	Including the maternal heterozygous site next to centromere
5	152,784	2,144,155	89	Including the first maternal heterozygous site next to the chromosome end
X	2,753,627	2,881,011	6	Including the first maternal heterozygous site next to the chromosome end

Acknowledgements

This study was supported by Key Laboratory Project in Shenzhen, CXB200903110066A and CXB201108250096A. We sincerely thank our colleagues in BGI for sequencing.

References

1. Li R, Yu C, Li Y, Lam T-W, Yiu S-M, Kristiansen K, Wang J: **SOAP2: an improved ultrafast tool for short read alignment.** *Bioinformatics* 2009, **25:**1966-1967.

2. Li R, Li Y, Fang X, Yang H, Wang J, Kristiansen K, Wang J: **SNP detection for massively parallel whole-genome resequencing.** *Genome Res* 2009, **19:**1124-1132.

3. Browning BL, Browning SR: **A unified approach to genotype imputation and haplotype-phase inference for large data sets of trios and unrelated individuals.** *Am J Hum Genet* 2009, **84:**210-223.

4. Rabiner LR: **A tutorial on hidden Markov models and selected applications in speech recognition.** *Proceedings of the IEEE* 1989, **77:**257-286.

5. **R: A Language and Environment for Statistical Computing.** R Foundation for Statistical Computing, 2008.

6. Chiang DY, Getz G, Jaffe DB, O'Kelly MJ, Zhao X, Carter SL, Russ C, Nusbaum C, Meyerson M, Lander ES: **High-resolution mapping of copy-number alterations with massively parallel sequencing.** *Nat Methods* 2009, **6:**99-103.

7. Lewin B. *Gene IX*. Burlington, MA: Jones and Bartlett Publishers, 2008.

8. Lo YMD, Chan KCA, Sun H, Chen EZ, Jiang P, Lun FMF, Zheng YW, Leung TY, Lau TK, Cantor CR, Chiu RWK: **Maternal plasma DNA sequencing reveals the genome-wide genetic and mutational profile of the fetus.** *Sci Transl Med* 2010, **2**, 61ra91.
